# PBAC: A pathway‐based attention convolution neural network for predicting clinical drug treatment responses

**DOI:** 10.1111/jcmm.18298

**Published:** 2024-04-29

**Authors:** Dexun Deng, Xiaoqiang Xu, Ting Cui, Mingcong Xu, Kunpeng Luo, Han Zhang, Qiuyu Wang, Chao Song, Chao Li, Guohua Li, Desi Shang

**Affiliations:** ^1^ The First Affiliated Hospital, Cardiovascular Lab of Big Data and Imaging Artificial Intelligence, Hengyang Medical School University of South China Hengyang Hunan China; ^2^ Hunan Provincial Key Laboratory of Multi‐omics And Artificial Intelligence of Cardiovascular Diseases University of South China Hengyang Hunan China; ^3^ School of Computer University of South China Hengyang Hunan China; ^4^ The First Affiliated Hospital, Institute of Cardiovascular Disease, Hengyang Medical School University of South China Hengyang Hunan China; ^5^ Department of Cardiology, The First Affiliated Hospital, Hengyang Medical School University of South China Hengyang China; ^6^ Department of Gastroenterology and Hepatology Second Affiliated Hospital of Harbin Medical University Harbin Heilongjiang China; ^7^ Department of Biochemistry and Molecular Biology, School of Basic Medical Sciences, Hengyang Medical School University of South China Hengyang Hunan China; ^8^ Department of Cell Biology and Genetics, School of Basic Medical Sciences, Hengyang Medical School University of South China Hengyang Hunan China; ^9^ Department of Anesthesiology The First Affiliated Hospital of University of South China Hengyang PR China; ^10^ Department of Pathophysiology, Key Laboratory for Arteriosclerology of Hunan Province, MOE Key Lab of Rare Pediatric Diseases, Hengyang Medical School Institute of Cardiovascular Disease, Hunan International Scientific and Technological Cooperation Base of Arteriosclerotic Disease, University of South China Hengyang Hunan China

**Keywords:** clinical treatment, deep learning, drug treatment response, interpretability

## Abstract

Precise and personalized drug application is crucial in the clinical treatment of complex diseases. Although neural networks offer a new approach to improving drug strategies, their internal structure is difficult to interpret. Here, we propose PBAC (Pathway‐Based Attention Convolution neural network), which integrates a deep learning framework and attention mechanism to address the complex biological pathway information, thereby provide a biology function‐based robust drug responsiveness prediction model. PBAC has four layers: gene‐pathway layer, attention layer, convolution layer and fully connected layer. PBAC improves the performance of predicting drug responsiveness by focusing on important pathways, helping us understand the mechanism of drug action in diseases. We validated the PBAC model using data from four chemotherapy drugs (Bortezomib, Cisplatin, Docetaxel and Paclitaxel) and 11 immunotherapy datasets. In the majority of datasets, PBAC exhibits superior performance compared to traditional machine learning methods and other research approaches (area under curve = 0.81, the area under the precision‐recall curve = 0.73). Using PBAC attention layer output, we identified some pathways as potential core cancer regulators, providing good interpretability for drug treatment prediction. In summary, we presented PBAC, a powerful tool to predict drug responsiveness based on the biology pathway information and explore the potential cancer‐driving pathways.

## 1 INTRODUCTION

Chemotherapy and immunotherapy are fundamental methods in cancer treatment. Chemotherapy, with its profound history, is a foundational therapy for cancer, while immunotherapy represents a novel approach to cancer treatment.^1^, ^2^ Both of these treatments are essential in clinical oncology. Nevertheless, they share a common limitation: they exhibit significant heterogeneity in treatment responses, with numerous patients failing to respond and encountering severe side effects from the therapies.^3^ Therefore, identifying a patient's potential response to drug treatment is extremely important for precision medicine in clinical practice.

A major challenge in the research of drug therapeutic response prediction is its practical application in the clinical context.^4^ Despite the significant advancements made by models such as developed by Ding et al.,^5^ AutoBorutaRF^6^ and DeepDR^7^ in predicting drug responses using omics data, translating these findings into real clinical practice remains a challenging task. Ideally, these models should be trained on in vivo data to ensure clinical relevance and applicability of their predictions.^8^ However, the omics data related to drug therapeutic responses predominantly come from in vitro studies of cell lines, with a limited availability of omics data obtained from actual clinical patients.^9^ Therefore, we need a model that not only performs well on cell line data but also demonstrates robust performance when utilized with clinical patient data.

Deep learning, as a powerful machine learning method,^10^ has demonstrated tremendous potential in predicting drug treatment responses. However, deep learning models still face challenges when applied in clinical practice. First, deep learning models typically require a large amount of data for training, while clinical patient data is often limited. Second, deep learning models are often considered as ‘black boxes’ with a lack of interpretability, which is unacceptable in clinical decision‐making. Therefore, exploring ways to extend deep learning models to address these issues is an important research direction. In recent years, some researchers have proposed methods to enhance the interpretability of deep learning models. For instance, the introduction of attention mechanisms^11^ allows the model to focus on important features relevant to the prediction task,^12^ thereby improving interpretability. Additionally, some researchers have attempted to incorporate domain knowledge and prior information into deep learning models to enhance their interpretability and generalization ability. For example, integrating biological pathway information can help explain the biological processes involved in predictions of the model.^13^

Acknowledging the common challenges of limited clinical applicability in models based on cell line data and the lack of biological interpretability often observed in deep learning models, we propose an interpretable model called pathway‐based attention convolution (PBAC) for the prediction of responses to clinical treatment. Specifically, we introduced a layer of pathway nodes and their connections to input gene nodes, assigning weights to pathway nodes before convolution. This framework presents two distinct advantages: first, it provides interpretability, enabling users to comprehend the crucial pathways involved in delineating a drug's response features. The second benefit is that even when models are trained on baseline data, they demonstrate exceptional performance when tested against clinical data. We conducted a comprehensive evaluation of various drug sensitivity and immunotherapy datasets, including cell line data related to four distinct chemotherapeutic agents (Bortezomib, Cisplatin, Docetaxel and Paclitaxel) along with their corresponding clinical datasets, as well as 11 unique datasets associated with immunotherapy. Our model showed superior performance across most of the datasets, outperforming traditional machine learning and deep learning methods. Additionally, through pathway scoring, we identified several biological pathways relevant to disease treatment. Overall, our model provides biological interpretability for predicting drug response and immunotherapy response, effectively bridging the gap between cell line data to clinical data.

## 2 MATERIALS AND METHODS

### 2.1 Datasets

To comprehensively evaluate the prediction performance of the proposed method, as shown in Table 1, we collected sensitivity data for four drugs (i.e. Bortezomib, Cisplatin, Docetaxel and Paclitaxel) from Genomics of Drug Sensitivity in Cancer (GDSC)^14^ database (www.cancerRxgene.org) and clinical patient data for these four drugs (GSE9782,^15^ GSE18864,^16^ GSE6434^17^ and GSE22513^18^) from the Gene Expression Omnibus (GEO) database (http://www.ncbi.nlm.nih.gov/geo/).^19^ To further assess the performance of PBAC, we collected seven sets of immunotherapy data (GSE100797, GSE35640, GSE19293, GSE91061, GSE78220, GSE106128 and GSE176307) from GEO, as well as four other published datasets (IMvigor210,^20^ PRJNA482620,^21^ Liu et al^22^ and phs000452^22^) as shown in Table 2.

**TABLE 1:** Drug sensitivity data includes cell line and clinical patient data in this study.

Datasets	Drug	Sample size	Resource
GDSC	Bortezomib	752	Cell line
GSE9782	Bortezomib	239	Clinical trial
GDSC	Cisplatin	764	Cell line
GSE18864	Cisplatin	24	Clinical trial
GDSC	Docetaxel	762	Cell line
GSE6434	Docetaxel	24	Clinical trial
GDSC	Paclitaxel	753	Cell line
GSE22513	Paclitaxel	28	Clinical trial

**TABLE 2:** 11 Immunotherapy datasets in this study.

Datasets	Drug	Sample size	Resource	Disease
GSE100797	ACT	25	Clinical trial	Melanoma
GSE35640	MAGE‐A3	56	Clinical trial	Melanoma
GSE19293	Melphalan	52	Clinical trial	Melanoma
GSE91061	Anti‐PD‐1	98	Clinical trial	Melanoma
GSE78220	Anti‐PD‐1	27	Clinical trial	Melanoma
GSE106128	DCS	35	Clinical trial	Melanoma
GSE176307	Anti‐PD‐1	88	Clinical trial	Bladder
IMvigor210	Anti‐PD‐L1	298	Clinical trial	Bladder
Liu et al	Nivolumab	121	Clinical trial	Melanoma
phs000452	Anti‐PD‐1	153	Clinical trial	Melanoma
PRJNA482620	Anti‐PD‐1	34	Clinical trial	G B M

### 2.2 PBAC: An interpretable pathway‐based attention convolution model

We propose a deep learning framework that can be utilized in clinical data for drug therapy response prediction. Specifically, the PBAC consists of four layers: gene‐pathway layer, attention layer, convolutional layer and fully connected layer.

#### 2.2.1 Gene‐pathway layer

The connections between the initial input layer and pathway layer are determined by the correlations between genes and pathways. A mask matrix, represented by M and with dimensions of 1358 × *n* (where, *n* represents the number of genes), was utilized to encode the relationship between gene nodes and pathway nodes. In this matrix, a value of 1 indicates the presence of an association between a gene and pathway node, while 0 signifies the lack of such an association. This process can be illustrated as:

$$X_{m}=X\times M$$

where $X_{m}$ represents the filtered gene expression data, *M* is the gene‐pathway connection matrix and X is the original gene expression data.

#### 2.2.2 Attention layer

In our model, we integrate an attention mechanism to determine and evaluate the importance of different biological pathways. This mechanism initially projects the features of each pathway into a latent space using a dense layer, followed by a tanh activation function. Then, these projected features are re‐mapped to the original feature space through another dense layer, producing attention scores for each specific pathway. These scores are then converted into weights using a softmax function, guaranteeing that the total sum of the weights across all pathways equals 1. This can be represented as:

$$W_{a}=\text{softmax}\;\left(W_{2}\times \tanh\;\left(W_{1}\times X_{m}\right)\right)$$

$$X_{a}=X_{m}\times W_{a}$$

where $X_{a}$ represents the output from the attention layer, $W_{1}$ and $W_{2}$ are the weights of the fully connected layers and $W_{a}$ denotes the computed attention weights.

#### 2.2.3 Convolutional layer and fully connected layer

We utilize a one‐dimensional convolutional layer to process the output from the attention mechanism. The objective of this layer's design is to capture and learn local correlations among different pathway features. Specifically, our convolutional layer is configured with a kernel size of 3, a stride of 1, and padding of 1. The fully connected layer is employed for the final classification task. It transforms the features extracted by the convolutional layer into the target class space, thereby generating the probabilities for each category. Mathematically, the operations of the convolutional layer and fully connected layer can be represented as:

$$X_{c}=\text{Conv}\;\left(X_{a}\right)$$

$$Y=\text{softmax}\;\left(W_{f}\times X_{c}+b\right)$$

where $X_{c}$ is the output from the convolutional layer, Y represents the output probabilities for each category, $W_{f}$ denotes the weights of the fully connected layer and b signifies the bias term.

### 2.3 Evaluation method

For drug sensitivity data related to cell lines, we classify the cell lines based on the Maximum Screening Concentration (Max Conc) provided in the GDSC database. If the IC50 value of a cell line exceeds the Max Conc, it is labelled as insensitive; otherwise, it is deemed sensitive. The Max Conc values for Bortezomib, Cisplatin, Docetaxel and Paclitaxel are 0.02, 8.00, 0.125 and 0.102, respectively. In all clinical data analysed in this study, patients with stable disease and progressive disease were classified as non‐responders, while those demonstrating either partial response or complete response were identified as responders.

We use the Area Under the Receiver Operating Characteristic curve (AUROC) and Area Under the Precision‐Recall Curve (AUPRC) to evaluate the predictive performance of the model. Specifically, to further illustrate the predictive performance of the model, we adopted effect size in drug sensitivity prediction. The effect size was calculated by taking the mean difference in the predicted IC50 z‐scores between responders and non‐responders.^23^

### 2.4 Statistical analysis

The effect size was computed as the mean difference in predicted IC50 z‐scores of responders versus non‐responders. The *p*‐value was calculated using the Wilcoxon rank sum (Mann–Whitney) test. The analysis was conducted in Python 3.7. For the immune infiltration analysis, we utilized the Cibersort package in R 4.1 and employed the Mann–Whitney–Wilcoxon test. In all statistical analyses, a significance level of *p* < 0.05 was set.

## 3 RESULTS

**FIGURE 1:**
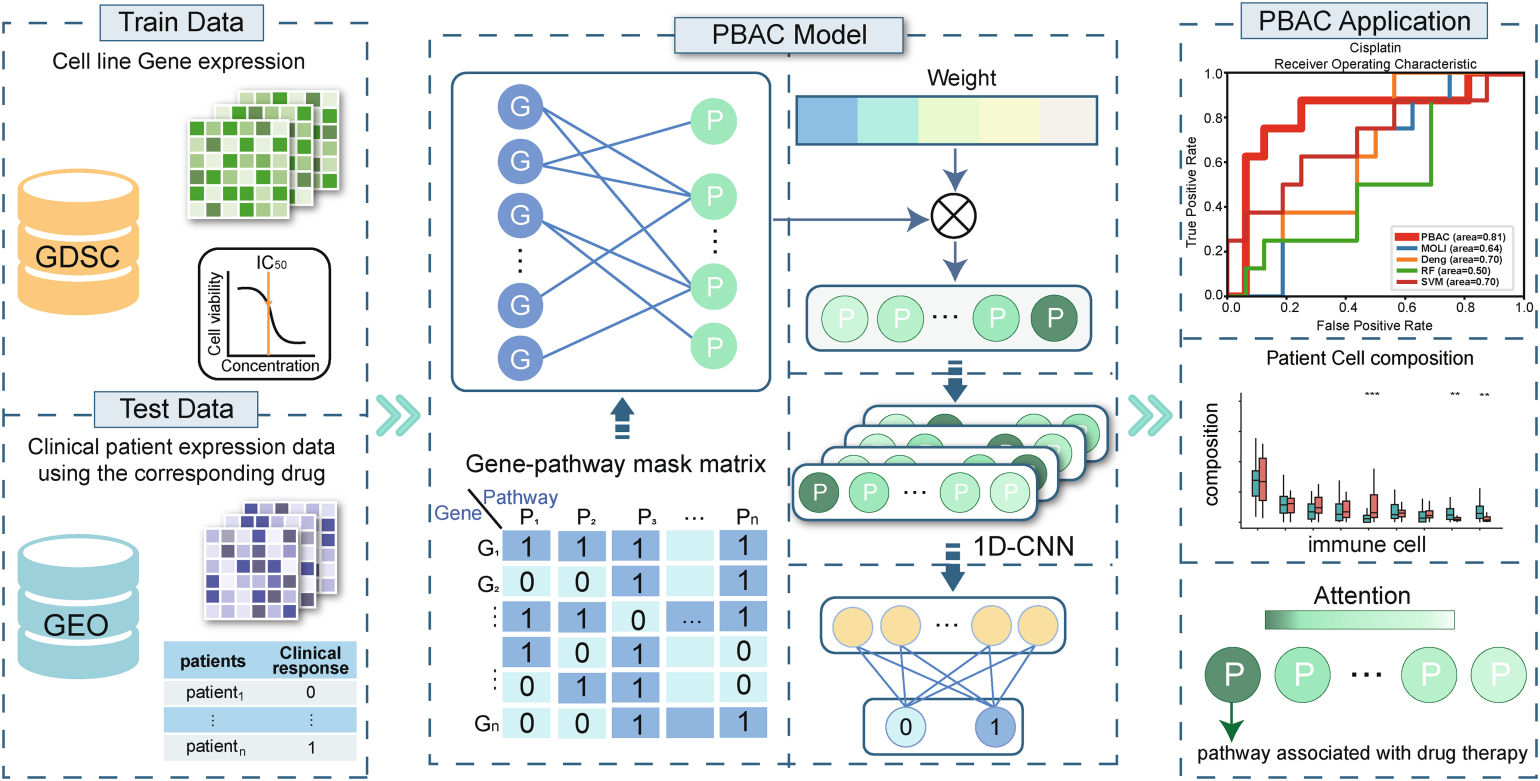
The overview of PBAC for drug therapy prediction framework. The gene expression data is processed through a gene‐pathway mask layer, an attention mechanism, and convolution for classification of the prediction results. Furthermore, the model undergoes evaluation and interpretability analysis.

As shown in Figure 1, we trained PBAC on GDSC cell lines screened with chemotherapeutic agents. The input, consisting of gene expression data, undergoes initial processing through a gene‐pathway masking layer. This layer functions to eliminate genes not associated with the critical pathways involved in the disease under investigation. Subsequently, an attention mechanism is utilized to prioritize pathway features that significantly impact the predictive outcome. Following this, convolution operations are applied for robust feature extraction. Ultimately, the model performs a classification prediction, offering valuable insights for disease prognosis or diagnosis. We assessed the model using clinical patient data related to specific drugs and successfully integrated immunotherapy data into the model. Based on the model's classification results, we conducted immune infiltration analysis. Additionally, using the attention scores from the model, we identified pathways that are highly correlated with disease treatment.^24^

### 3.1 Chemotherapy drug response prediction

**FIGURE 2:**
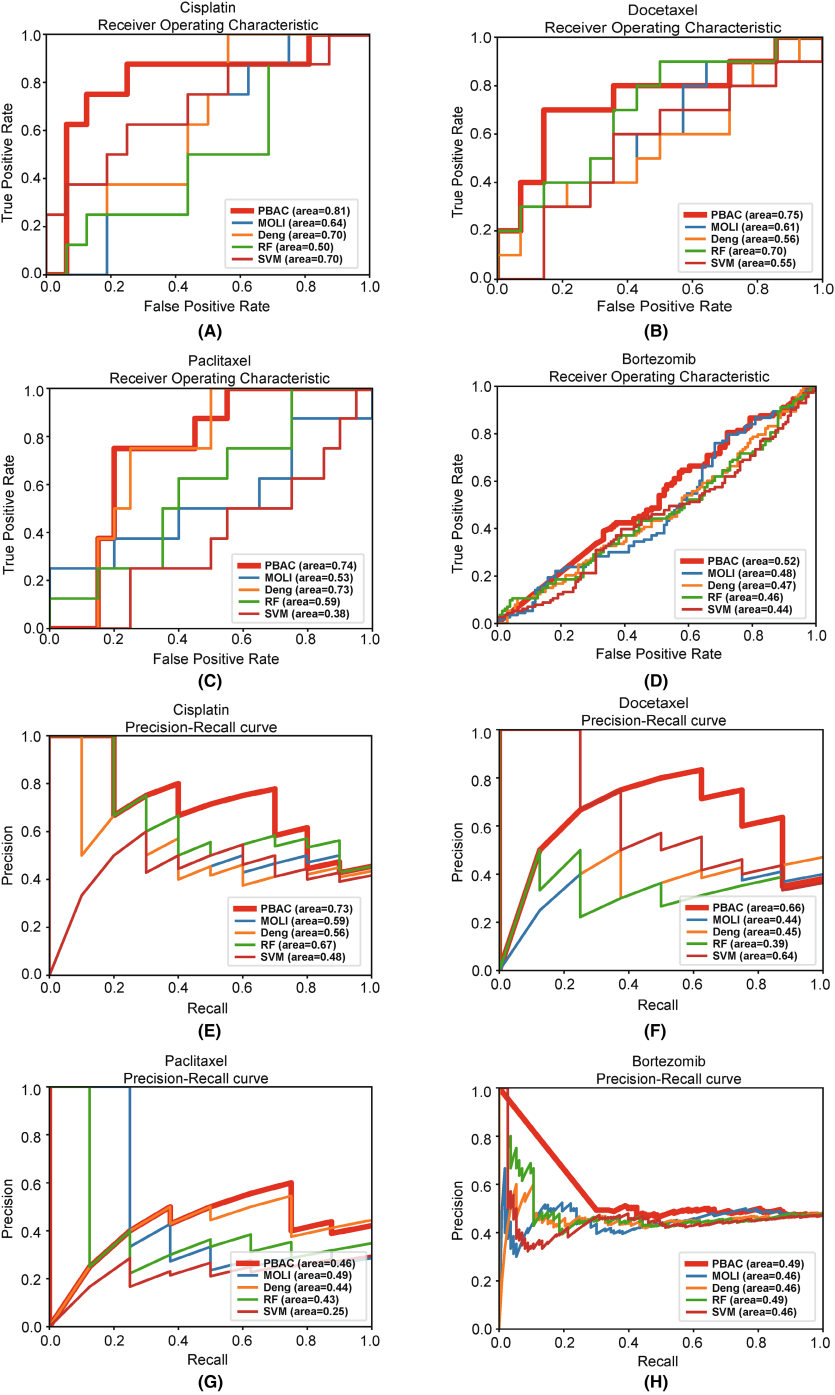
The comparison of ROC curves and the precision‐recall curves of PBAC model in clinical data with traditional machine learning and existing research methods. (A–D) Are the ROC curves of four chemotherapy drugs. (E–H) Are the precision‐recall curve of four chemotherapy drugs.

**FIGURE 3:**
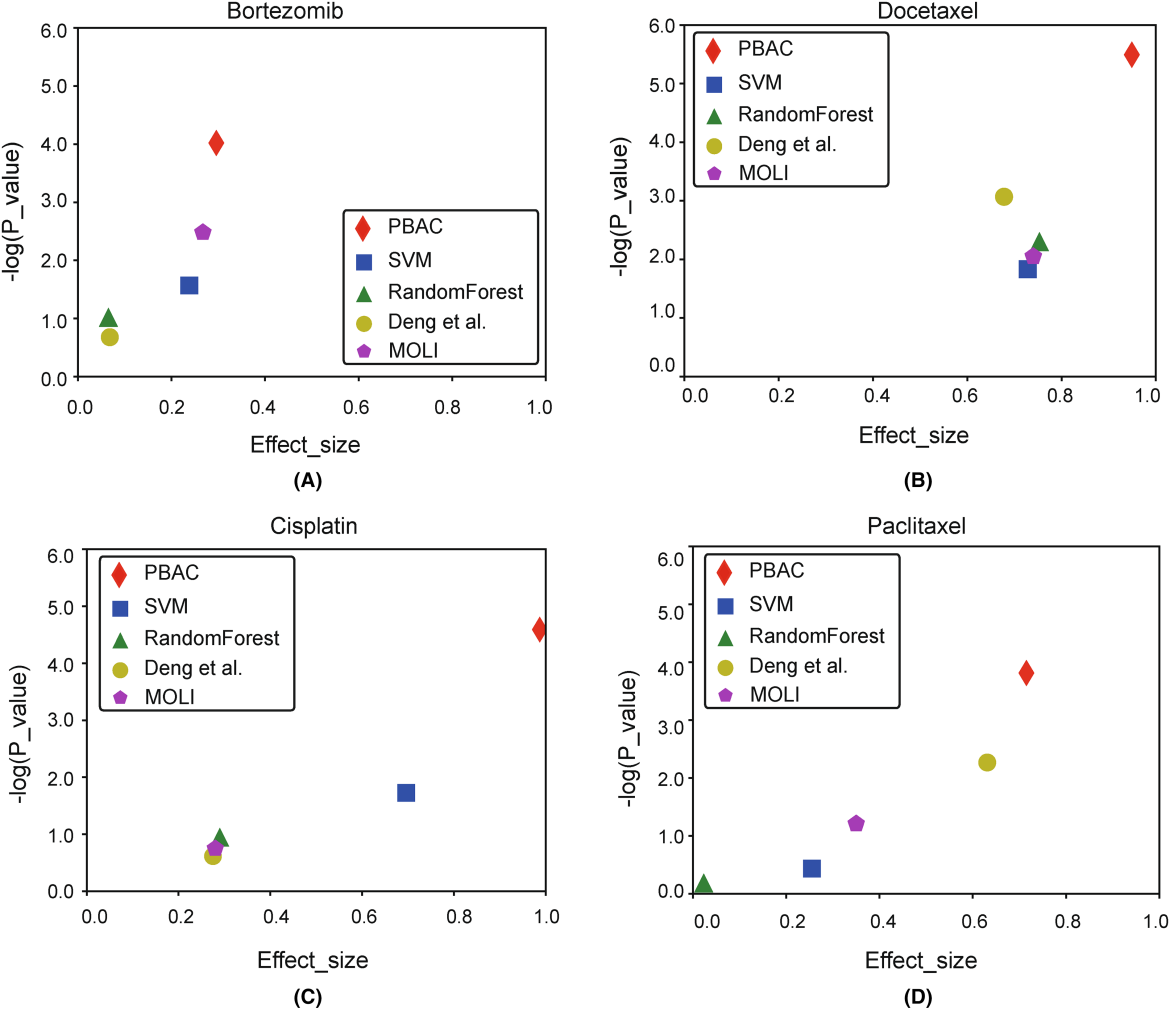
Volcano plots of effect size (difference of mean IC50 between responders and non‐responders) and *p*‐values (Wilcox test) of each learning algorithm.

In the current study, we trained our model using cell line data associated with four chemotherapy drugs: Bortezomib, Cisplatin, Docetaxel and Paclitaxel. Subsequently, we tested the model using clinical patient data specific to these four drugs. The performance of our model was evaluated based on three key metrics: The AUROC, AUPRC and effect size. We compare the performance of our model with MOLI^8^ and the method proposed by Deng et al.^13^ Additionally, we contrasted our approach with conventional machine learning methods, namely Random Forest and Support Vector Machine. As shown in Figure 2A–H, our model delivers reliable predictions of chemotherapy drug responses, surpassing previous research methods and conventional machine learning approaches. To further demonstrate the predictive capability of the model, we utilized effect size in drug sensitivity prediction. We adjusted the model's output to IC50 values, avoiding the binary classification into 0 and 1 during label processing for the cell line data. Subsequently, we calculated the effect size based on the predicted IC50 values and the response categories of clinical patients. Furthermore, the effect size of the PBAC model was superior to other methods across all four drugs (Figure 3A–D).

### 3.2 Ablation study in the PBAC model

**FIGURE 4:**
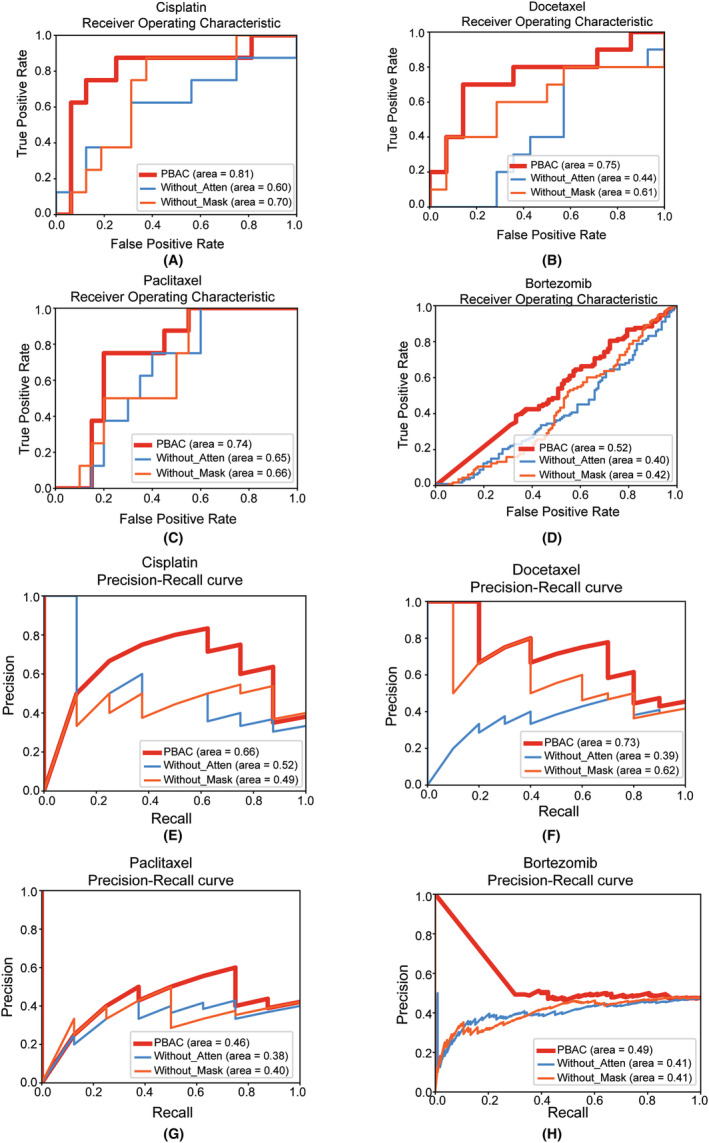
The comparison of AUC and Area Under the Precision‐Recall Curve (AUPRC) in PBAC's Ablation experiment. (A–D) are the ROC curve of ablation experiments for four different drugs, while (E–H) represent the precision‐recall curves of four different drugs.

In the ablation experiment, we investigated the individual contributions of the masking layer and attention layer to the overall performance of the PBAC model. By systematically removing each of these components from the model and observing the subsequent effect on its predictive performance, we were able to quantify their respective roles in chemotherapy drug response prediction. Figure 4A–D show the ROC curves of the PBAC model after removing the gene pathway masking module and attention module. According to the trends of the ROC curves and AUC values, it is evident that the predictive performance of the PBAC model for the efficacy of four chemotherapy drugs significantly decreases in the ablation experiment. Similarly, from the precise recall curves of the PBAC model in the ablation experiment (Figure 4E–H), we find that AUPRC also decreases significantly after removing each module individually. This indicates the critical role of the masking layer in managing input variations and reducing the impact of irrelevant features, while the attention layer effectively focuses on key predictive features during the learning process.

### 3.3 Immunotherapy response prediction

Over the past few years, immunotherapy has significantly revolutionized the clinical treatment landscape for cancer patients.^25^ Despite the substantial clinical benefits offered by immunotherapy, a notable limitation is that only a minority of patients (~30% in solid tumours) exhibit responsiveness to this treatment. Furthermore, there's a potential for toxicity following immunotherapy.^26^ Therefore, we used the PBAC model to predict treatment responses in the immunotherapy datasets. Our model was assessed utilizing 11 distinct immunotherapy datasets, comprising over 1000 patient samples across various cancer types and immunotherapy modalities. Table 2 provides detailed information. Specifically, we executed two types of predictions: (1) within‐study predictions, where both training and test datasets were sourced from a single cohort and (2) across‐study predictions, where two distinct datasets were deployed as the training and test sets.^27^

**FIGURE 5:**
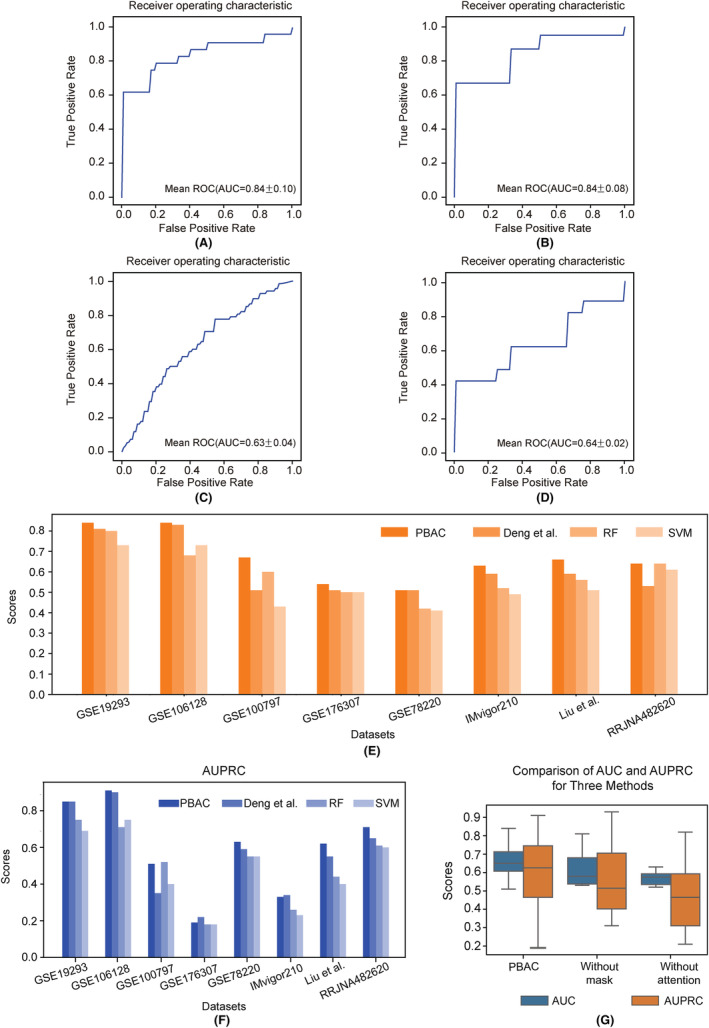
The ROC curve of the immune therapy datasets in within‐study performing five‐fold cross‐validation on PBAC. (A–D) represent the ROC curves of performance on the GSE19293, GSE106128, IMvigor210 and PRJNA482620, respectively. (E) The AUC performance of PBAC and other methods across eight datasets in within‐study. (F) The Area Under the Precision‐Recall Curve (AUPRC) performance of PBAC and other methods across eight datasets in within‐study. (G) Performance comparison of ablation experiments and PBAC in eight datasets.

In the within‐study predictions, we conducted five‐fold cross validation on each dataset (Figure 5A–D), and calculated the average AUC (Figure 5E) and AUPRC (Figure 5F) to assess the performance of the model. Overall, among the 11 immunotherapy datasets we collected, our model outperformed both Deng et al.^13^ method and traditional machine learning methods in eight datasets. During the cross‐validation process, datasets GSE19293 and GSE106128 demonstrated exceptional performance with AUC values exceeding 0.84. Furthermore, their respective AUPRC reached notable scores of 0.85 and 0.91, respectively. In dataset GSE78220, although the AUC values of PBAC and Deng's method are equal, PBAC demonstrates a higher AUPRC. To show the significant influence of the masking layer and attention layer on predicting immunotherapy response, similar to the process of predicting chemotherapy response, we conducted ablation experiments on these high‐performing datasets. The removal of the mask layer and attention layer results in varying degrees of decline in both AUC and AUPRC (Figure 5G).

**FIGURE 6:**
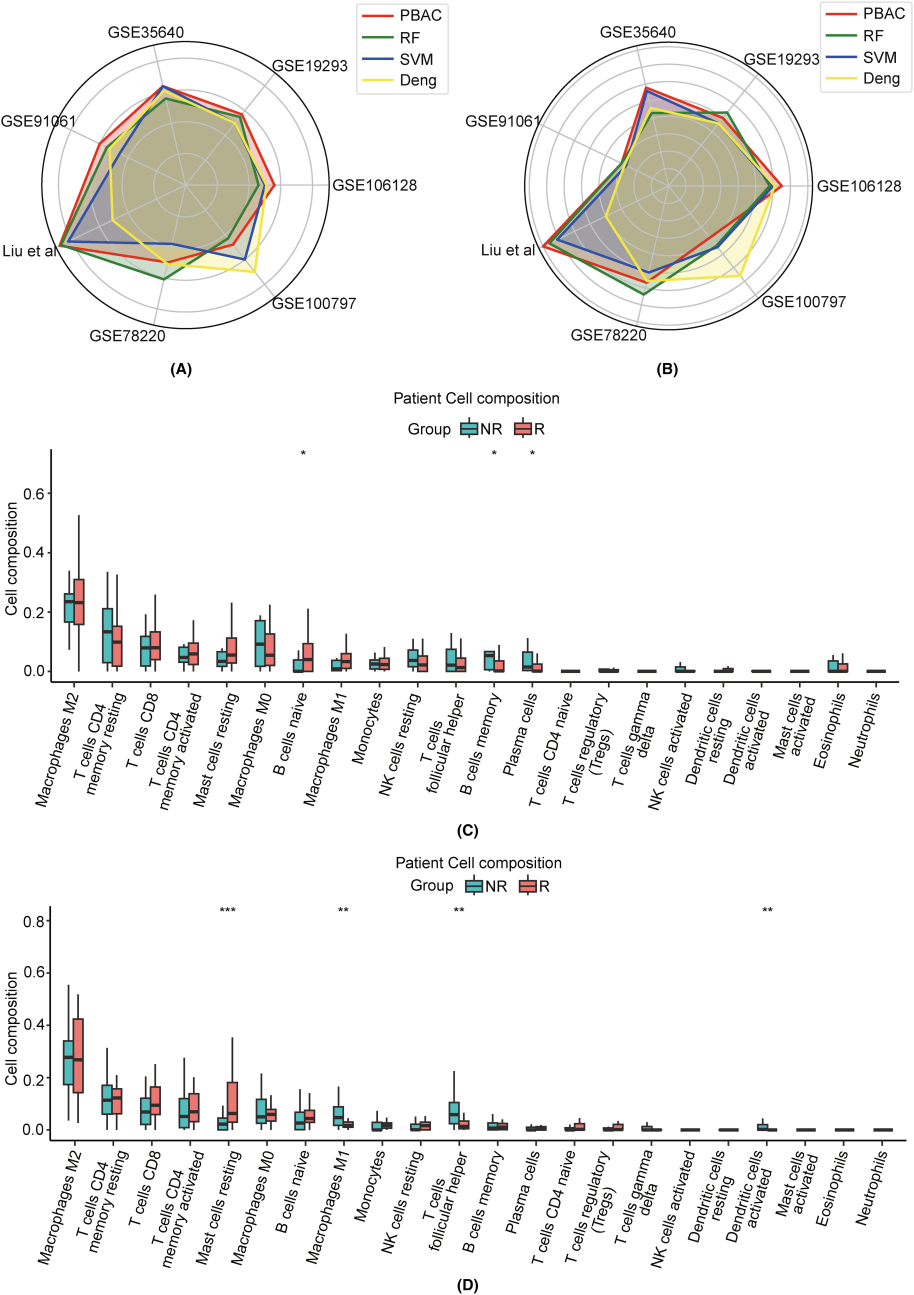
(A) The comparison of AUC between PBAC and various methods across different datasets in across‐study. (B) The comparison of Area Under the Precision‐Recall Curve (AUPRC). (C, D) In the dataset Liu et al. and GSE91061, the immune cell components of the two categories were analysed based on the classification results of PBAC (dividing patients into responders (R) and non‐responders (NR)). * represents *p* < 0.05, ** represents *p* < 0.01, and *** represents *p* < 0.001.

For the across‐study predictions, we chose to focus on melanoma due to its prevalence in our datasets. The phs000452 dataset, with the largest sample size (153 samples) among the melanoma datasets, was selected as the training set. The remaining seven datasets were used as the test sets. PBAC demonstrated significant superiority over other models in five of these datasets (Figure 6A,B). Our model shows superior performance in datasets with larger sample sizes. In Liu et al. dataset with 121 samples, PBAC performs the best (AUC = 0.80), whereas in datasets GSE78220 (27 samples) and GSE100797 (25 samples) with sample sizes less than 30, PBAC performs worse than other models. To validate our prediction results, we employed the CIBERSORT algorithm^28^ to analyse immune cell types in gene expression data, and integrated the results of CIBERSORT with the group information we predicted. Subsequently, we performed a Wilcoxon test for each type of immune cell between different groups and visualized the results using box plots. In the grouping based on the PBAC model, we observed significant variances in immune cell types between the response group and non‐response group. For instance, in Liu et al. dataset, notable differences were found in the proportions of B cells naive, B cells memory and plasma cells among different groups (Figure 6C). In the GSE91061 dataset, significant distinctions were noted in the proportions of mast cells resting, macrophages M1 and T cells follicular helper, among others (Figure 6D). Our study indicates that, even in the cross‐dataset prediction, PBAC retains a consistent level of performance and outperforms alternative methods.

### 3.4 Biological interpretability of the PBAC model

**FIGURE 7:**
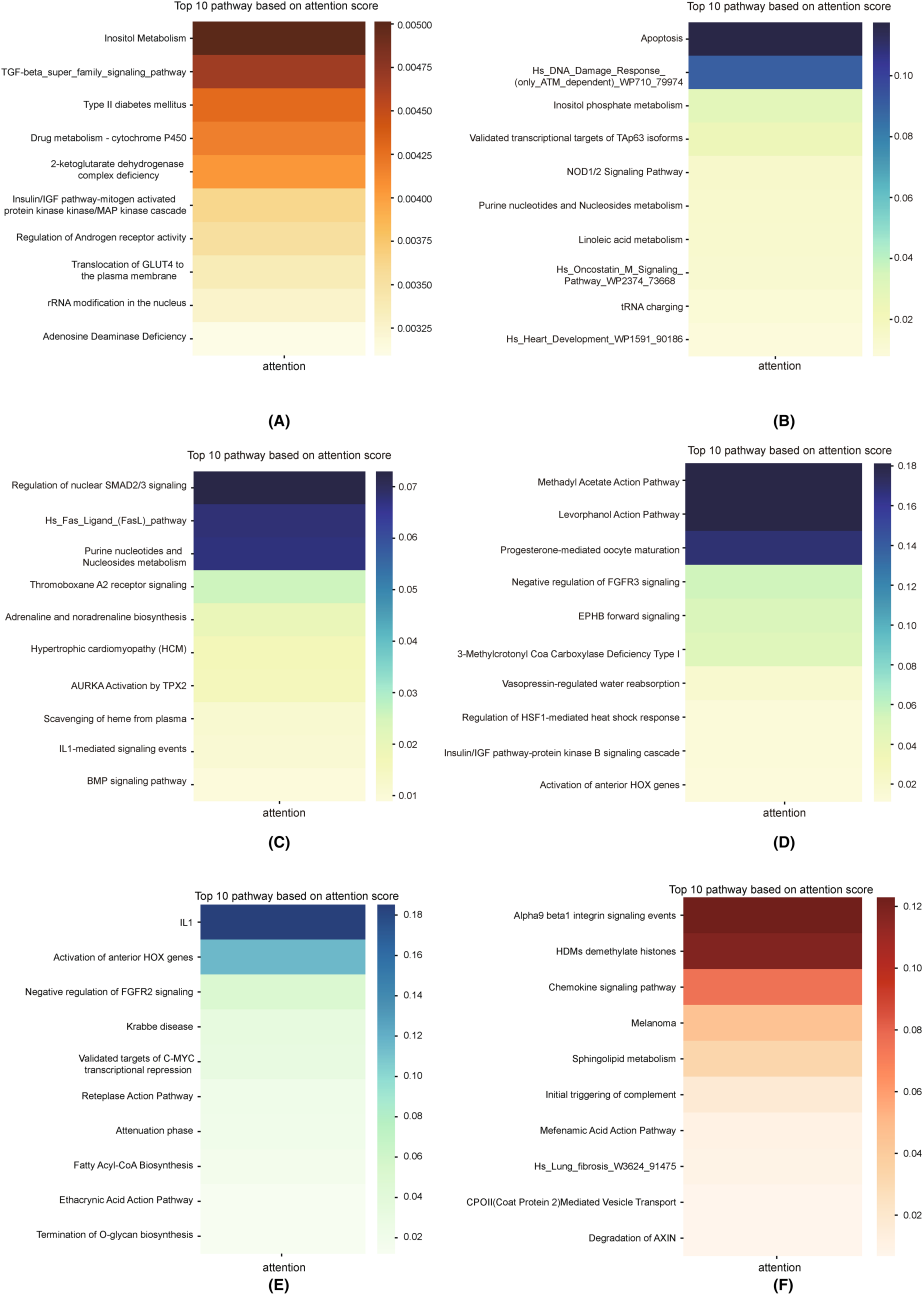
The top 10 pathways ranked by attention scores obtained from training the model on the datasets. The heatmaps for the top 10 pathways of (A–F) correspond respectively to Cisplatin, GSE19293, GSE106128, Liu et al, IMvigor210 and PBJNA482620.

Advantage of PBAC lies in its interpretability, coupled with its ability to identify significant pathway features for predicting treatment responses. Identifying pathways related to drug response is crucial for comprehending the mechanism of drug action. To achieve this, we ranked the importance of pathways in predicting drug treatment responses based on the attention scores obtained from the model. For instance, in the pathway ranking of the cisplatin drug dataset GSE18864 (Figure 7A), we identified a cytochrome P450 related pathway (i.e. Drug metabolism–cytochrome P450). Cytochrome P450 (CYP450) plays a crucial role in drug metabolism, with studies showing that this pathway is highly associated with anti‐breast cancer drugs, particularly serving as an important drug target in breast cancer treatment.^29^ As the major anti‐cancer drug for breast cancer, the drug is metabolized to its active metabolites by the cytochrome P450.^30^ In the melanoma immunotherapy dataset GSE19293, we identified a cell apoptosis pathway related to melanoma treatment (Figure 7B). Dysregulation of the apoptosis pathway hinders the treatment of melanoma.^31^ Recent research also indicated that apoptosis induced by anticancer drugs may be a predictor of drug sensitivity.^32^ For example, proteasome inhibitors alone, or in combination with other drugs, efficiently induce apoptosis in melanoma cells and the apoptosis status is the indicator of drug efficiency.^33^ In the top‐ranked pathways identified in the GSE19293 dataset, we also discovered the purine metabolism pathway. The enzyme complex within the purine metabolism pathway could serve as a potential target for tumour treatment.^34^ Purines are basic components of nucleotides in cell proliferation and the impaired purine metabolism is associated with the progression of cancer. The major anti‐cancer drug‐rapamycin is the purinosome formation and purine metabolism regulator, the purine‐related metabolism process is served as the target of rapamycin.^35^ Remarkably, within the top 10 pathways of the GSE106128 dataset (Figure 7C), we also identified the presence of pathway purine metabolism. Due to individual differences, the top 10 pathways obtained in different datasets were also different. However, we still found some relevant pathways in other melanoma datasets. For example, in Liu et al. dataset, we identified the ‘Regulation of HSF1‐mediated heat shock response’ pathway (Figure 7D), which is related to the human melanoma phenotype.^36^ Besides, the expression of the IL1 pathway, which ranks first in attention scores in the IMvigor210 bladder cancer dataset (Figure 7E), may be associated with the invasive capability of bladder cancer cell lines.^37^ In the top 10 pathways of the glioblastoma multiforme (GBM) dataset (Figure 7F), the chemokine signalling pathway is associated with tumour progression, and in GBM, the chemokine receptor signalling pathway is activated.^38^ In summary, PBAC is capable of inferring the importance of specific biological pathways in treatment responses.

## 4 DISCUSSION

Chemotherapy and immunotherapy are significant methods of tumour treatment, but both therapies exhibit high heterogeneity in treatment responses and are associated with severe side effects. Consequently, precision treatment is increasingly important in clinical practice. However, the lack of Omics clinical data makes it challenging to accurately predict treatment responses based solely on such data. Moreover, most existing models for predicting treatment responses are essentially black boxes,^39^ lacking interpretability in a biological sense. Pathways, as crucial components of biological processes, can aid in understanding the mechanisms of disease progression and treatment strategies. Therefore, we have incorporated information on disease‐associated pathways to guide the connections within our model, providing it with biological interpretability. Furthermore, we utilized an attention mechanism to assign higher weights to pathways that are more important for predicting outcomes.^11^, ^40^ Through analysing the weights, we can identify pathways that play crucial roles in specific drug treatments. Due to the large number of pathways, the features are also high‐dimensional. To further extract features, reduce the model's training parameters and prevent overfitting, we incorporated convolutional layers to enhance the model performance. We trained the PBAC model on the datasets of Bortezomib, Cisplatin, Docetaxel and Paclitaxel chemotherapy drugs, and evaluated its performance on corresponding clinical data. Our model significantly outperformed MOLI,^8^ the method proposed by Deng et al.,^13^ as well as traditional machine learning methods such as random forests and support vector machines. To further validate the stability and clinical utility of the model, we assessed the performance of PBAC on 11 immunotherapy datasets, with results showing that our model performed better in most datasets.

More importantly, PBAC managed to capture some complex processes of biological interaction based on the attention mechanism. In chemotherapy, we identified the breast cancer‐related pathway Cytochrome P450 (CYP450) in the cisplatin treatment data. This pathway plays a crucial role as a drug target in breast cancer treatment,^30^ indicating that our model can identify important pathways for specific drugs in disease treatment, helping us understand the mechanism of drug action. Additionally, numerous insights were gained in immunotherapy. For example, by ranking pathway attention scores in the melanoma dataset GSE19293, we identified several pathways closely associated with melanoma. Furthermore, in the melanoma dataset GSE106128, we found the same pathways as in GSE19293, validating the stability of our model and demonstrating that PBAC can identify important pathways related to specific diseases. To validate the interpretability of our model in different types of cancer, we generated attention scores for bladder cancer and glioblastoma datasets. As expected, we identified relevant key pathways within their top 10 pathways. For example, in the bladder cancer dataset IMvigor210, we identified the IL1 pathway associated with the invasive ability of bladder cancer cell lines. In the GBM dataset, we found the activation of the Chemokine signalling pathway related to tumour progression. This demonstrates that PBAC can identify pathways relevant to different types of diseases, indicating a high level of generalizability.

Although our study has some limitations and requires a more comprehensive dataset of chemotherapy and immunotherapy to further enhance the robustness of the model, the continuous development of big data offers a solution. With the integration of additional datasets into research, we expect to overcome these challenges. In summary, we have introduced an effective pathway‐based interpretable deep learning model that has the potential to aid in precision medication in clinical practice, alleviate the side effects of complex mechanism drug treatments and address the issue of non‐responsiveness to clinical drug therapies.

## AUTHOR CONTRIBUTIONS

**Dexun Deng:** Data curation (lead); formal analysis (lead); methodology (equal); validation (lead); writing – original draft (lead). **Xiaoqiang Xu:** Data curation (equal); methodology (lead). **Ting Cui:** Data curation (equal); formal analysis (equal). **Mingcong Xu:** Formal analysis (equal). **Kunpeng Luo:** Formal analysis (equal); writing – review and editing (equal). **Han Zhang:** Formal analysis (equal). **Qiuyu Wang:** Data curation (equal); formal analysis (equal). **chao song:** Formal analysis (equal). **Chao Li:** Conceptualization (equal); writing – review and editing (equal). **Guohua Li:** Conceptualization (equal); writing – review and editing (equal). **desi shang:** Conceptualization (lead); writing – review and editing (lead).

## FUNDING INFORMATION

This work was supported by National Natural Science Foundation of China [62272211, 62272212]; Natural Science Foundation of Hunan Province [2023JJ30535, 2023JJ30547, 2023JJ40594, 2022JJ30502]; Research Foundation of the First Affiliated Hospital of University of South China for Advanced Talents [20210002‐1005 USCAT‐2021‐01]; China Postdoctoral Science Foundation [2019M661311]; 2020 the Scientific Research Project of Hunan Provincial Department of Education [20B493]; Hengyang Science and Technology Plan Project [202250045217].

## CONFLICT OF INTEREST STATEMENT

The authors declare no conflicts of interest.

## Data Availability

The data that support the findings of this study are available from the corresponding author upon reasonable request. The source codes for PBAC were developed in python 3.7 and are available at a GitHub: https://github.com/YuZhengM/PBAC.

## References

[1] Borgers JSW , Heimovaara JH , Cardonick E , et al. Immunotherapy for cancer treatment during pregnancy. Lancet Oncol. 2021;22(12):e550‐e561. doi:10.1016/s1470-2045(21)00525-8 34856152

[2] Qin SY , Zhang AQ , Cheng SX , Rong L , Zhang XZ . Drug self‐delivery systems for cancer therapy. Biomaterials. 2017;112:234‐247. doi:10.1016/j.biomaterials.2016.10.016 27768976

[3] Peng M , Mo Y , Wang Y , et al. Neoantigen vaccine: an emerging tumor immunotherapy. Mol Cancer. 2019;18(1):128. doi:10.1186/s12943-019-1055-6 31443694 PMC6708248

[4] Geeleher P , Zhang Z , Wang F , et al. Discovering novel pharmacogenomic biomarkers by imputing drug response in cancer patients from large genomics studies. Genome Res. 2017;27(10):1743‐1751. doi:10.1101/gr.221077.117 28847918 PMC5630037

[5] Ding MQ , Chen L , Cooper GF , Young JD , Lu X . Precision oncology beyond targeted therapy: combining omics data with machine learning matches the majority of cancer cells to effective therapeutics. Mol Cancer Res. 2018;16(2):269‐278. doi:10.1158/1541-7786.Mcr-17-0378 29133589 PMC5821274

[6] Xu X , Gu H , Wang Y , Wang J , Qin P . Autoencoder based feature selection method for classification of anticancer drug response. Front Genet. 2019;10:233. doi:10.3389/fgene.2019.00233 30972101 PMC6445890

[7] Chiu YC , Chen HH , Zhang T , et al. Predicting drug response of tumors from integrated genomic profiles by deep neural networks. BMC Med Genet. 2019;12:18. doi:10.1186/s12920-018-0460-9 PMC6357352 30704458

[8] Sharifi‐Noghabi H , Zolotareva O , Collins CC , Ester M . MOLI: multi‐omics late integration with deep neural networks for drug response prediction. Bioinformatics. 2019;35(14):i501‐i509. doi:10.1093/bioinformatics/btz318 31510700 PMC6612815

[9] Zhao N , Liu Y , Wei Y , et al. Optimization of cell lines as tumour models by integrating multi‐omics data. Brief Bioinform. 2017;18(3):515‐529. doi:10.1093/bib/bbw082 27694350

[10] Wang W , Zhang L , Sun J , Zhao Q , Shuai J . Predicting the potential human lncRNA‐miRNA interactions based on graph convolution network with conditional random field. Brief Bioinform. 2022;23(6):bbac463. doi:10.1093/bib/bbac463 36305458

[11] Chen Z , Zhang L , Sun J , Meng R , Yin S , Zhao Q . DCAMCP: a deep learning model based on capsule network and attention mechanism for molecular carcinogenicity prediction. J Cell Mol Med. 2023;27(20):3117‐3126. doi:10.1111/jcmm.17889 37525507 PMC10568665

[12] Wang T , Sun J , Zhao Q . Investigating cardiotoxicity related with hERG channel blockers using molecular fingerprints and graph attention mechanism. Comput Biol Med. 2023;153:106464. doi:10.1016/j.compbiomed.2022.106464 36584603

[13] Deng L , Cai Y , Zhang W , Yang W , Gao B , Liu H . Pathway‐guided deep neural network toward interpretable and predictive modeling of drug sensitivity. J Chem Inf Model. 2020;60(10):4497‐4505. doi:10.1021/acs.jcim.0c00331 32804489

[14] Yang W , Soares J , Greninger P , et al. Genomics of drug sensitivity in cancer (GDSC): a resource for therapeutic biomarker discovery in cancer cells. Nucleic Acids Res. 2013;41:D955‐D961. doi:10.1093/nar/gks1111 23180760 PMC3531057

[15] Mulligan G , Mitsiades C , Bryant B , et al. Gene expression profiling and correlation with outcome in clinical trials of the proteasome inhibitor bortezomib. Blood. 2007;109(8):3177‐3188. doi:10.1182/blood-2006-09-044974 17185464

[16] Silver DP , Richardson AL , Eklund AC , et al. Efficacy of neoadjuvant cisplatin in triple‐negative breast cancer. J Clin Oncol. 2010;28(7):1145‐1153. doi:10.1200/jco.2009.22.4725 20100965 PMC2834466

[17] Chang JC , Wooten EC , Tsimelzon A , et al. Patterns of resistance and incomplete response to docetaxel by gene expression profiling in breast cancer patients. J Clin Oncol. 2005;23(6):1169‐1177. doi:10.1200/jco.2005.03.156 15718313

[18] Bauer JA , Chakravarthy AB , Rosenbluth JM , et al. Identification of markers of taxane sensitivity using proteomic and genomic analyses of breast tumors from patients receiving neoadjuvant paclitaxel and radiation. Clin Cancer Res. 2010;16(2):681‐690. doi:10.1158/1078-0432.Ccr-09-1091 20068102 PMC2892225

[19] Barrett T , Wilhite SE , Ledoux P , et al. NCBI GEO: archive for functional genomics data sets—update. Nucleic Acids Res. 2013;41:D991‐D995. doi:10.1093/nar/gks1193 23193258 PMC3531084

[20] Mariathasan S , Turley SJ , Nickles D , et al. TGFβ attenuates tumour response to PD‐L1 blockade by contributing to exclusion of T cells. Nature. 2018;554(7693):544‐548. doi:10.1038/nature25501 29443960 PMC6028240

[21] Riaz N , Havel JJ , Makarov V , et al. Tumor and microenvironment evolution during immunotherapy with nivolumab. Cell. 2017;171(4):934‐949.e16. doi:10.1016/j.cell.2017.09.028 29033130 PMC5685550

[22] Liu D , Schilling B , Liu D , et al. Integrative molecular and clinical modeling of clinical outcomes to PD1 blockade in patients with metastatic melanoma. Nat Med. 2019;25(12):1916‐1927. doi:10.1038/s41591-019-0654-5 31792460 PMC6898788

[23] Sakellaropoulos T , Vougas K , Narang S , et al. A deep learning framework for predicting response to therapy in cancer. Cell Rep. 2019;29(11):3367‐3373.e4. doi:10.1016/j.celrep.2019.11.017 31825821

[24] Hu H , Feng Z , Lin H , et al. Modeling and analyzing single‐cell multimodal data with deep parametric inference. Brief Bioinform. 2023;24(1):bbad005. doi:10.1093/bib/bbad005 36642414

[25] Gide TN , Wilmott JS , Scolyer RA , Long GV . Primary and acquired resistance to immune checkpoint inhibitors in metastatic melanoma. Clin Cancer Res. 2018;24(6):1260‐1270. doi:10.1158/1078-0432.Ccr-17-2267 29127120

[26] Havel JJ , Chowell D , Chan TA . The evolving landscape of biomarkers for checkpoint inhibitor immunotherapy. Nat Rev Cancer. 2019;19(3):133‐150. doi:10.1038/s41568-019-0116-x 30755690 PMC6705396

[27] Kong J , Ha D , Lee J , et al. Network‐based machine learning approach to predict immunotherapy response in cancer patients. Nat Commun. 2022;13(1):3703. doi:10.1038/s41467-022-31535-6 35764641 PMC9240063

[28] Chen B , Khodadoust MS , Liu CL , Newman AM , Alizadeh AA . Profiling tumor infiltrating immune cells with CIBERSORT. Methods Mol Biol. 2018;1711:243‐259. doi:10.1007/978-1-4939-7493-1_12 29344893 PMC5895181

[29] Dehal SS , Brodie AM , Kupfer D . The aromatase inactivator 4‐hydroxyandrostenedione (4‐OH‐A) inhibits tamoxifen metabolism by rat hepatic cytochrome P‐450 3A: potential for drug‐drug interaction of tamoxifen and 4‐OH‐a in combined anti‐breast cancer therapy. Drug Metab Dispos. 1999;27(3):389‐394. 10064571

[30] Singh MS , Francis PA , Michael M . Tamoxifen, cytochrome P450 genes and breast cancer clinical outcomes. Breast. 2011;20(2):111‐118. doi:10.1016/j.breast.2010.11.003 21185724

[31] Singhal SS , Srivastava S , Mirzapoiazova T , Horne D , Awasthi S , Salgia R . Targeting the mercapturic acid pathway for the treatment of melanoma. Cancer Lett. 2021;518:10‐22. doi:10.1016/j.canlet.2021.06.004 34126193

[32] Gartel AL . Mechanisms of apoptosis induced by anticancer compounds in melanoma cells. Curr Top Med Chem. 2012;12(1):50‐52. doi:10.2174/156802612798919196 22280162

[33] Was H , Borkowska A , Bagues A , et al. Mechanisms of chemotherapy‐induced neurotoxicity. Front Pharmacol. 2022;13:750507. doi:10.3389/fphar.2022.750507 35418856 PMC8996259

[34] Zhao H , French JB , Fang Y , Benkovic SJ . The purinosome, a multi‐protein complex involved in the de novo biosynthesis of purines in humans. Chem Commun (Camb). 2013;49(40):4444‐4452. doi:10.1039/c3cc41437j 23575936 PMC3877848

[35] Yin J , Ren W , Huang X , Deng J , Li T , Yin Y . Potential mechanisms connecting purine metabolism and cancer therapy. Front Immunol. 2018;9:1697. doi:10.3389/fimmu.2018.01697 30105018 PMC6077182

[36] Toma‐Jonik A , Widlak W , Korfanty J , et al. Active heat shock transcription factor 1 supports migration of the melanoma cells via vinculin down‐regulation. Cell Signal. 2015;27(2):394‐401. doi:10.1016/j.cellsig.2014.11.029 25435429

[37] Schneider L , Liu J , Zhang C , et al. The role of Interleukin‐1‐receptor‐antagonist in bladder cancer cell migration and invasion. Int J Mol Sci. 2021;22(11):5875. doi:10.3390/ijms22115875 34070905 PMC8198563

[38] Alghamri MS , Banerjee K , Mujeeb AA , et al. Systemic delivery of an adjuvant CXCR4‐CXCL12 signaling inhibitor encapsulated in synthetic protein nanoparticles for glioma immunotherapy. ACS Nano. 2022;16(6):8729‐8750. doi:10.1021/acsnano.1c07492 35616289 PMC9649873

[39] Gao H , Sun J , Wang Y , et al. Predicting metabolite‐disease associations based on auto‐encoder and non‐negative matrix factorization. Brief Bioinform. 2023;24(5):bbad259. doi:10.1093/bib/bbad259 37466194

[40] Meng R , Yin S , Sun J , Hu H , Zhao Q . scAAGA: single cell data analysis framework using asymmetric autoencoder with gene attention. Comput Biol Med. 2023;165:107414. doi:10.1016/j.compbiomed.2023.107414 37660567

